# ABT-199 (venetoclax) and BCL-2 inhibitors in clinical development

**DOI:** 10.1186/s13045-015-0224-3

**Published:** 2015-11-20

**Authors:** Shundong Cang, Chaitanya Iragavarapu, John Savooji, Yongping Song, Delong Liu

**Affiliations:** Department of Oncology, The Henan Province People’s Hospital, Zhengzhou, China; Department of Medicine, Westchester Medical Center and New York Medical College, Valhalla New York, 10595 USA; Henan Cancer Hospital and the Affiliated Cancer Hospital of Zhengzhou University, Zhengzhou, China

## Abstract

With the advent of new agents targeting CD20, Bruton’s tyrosine kinase, and phosphoinositol-3 kinase for chronic lymphoid leukemia (CLL), more treatment options exist than ever before. B-cell lymphoma-2 (BCL-2) plays a major role in cellular apoptosis and is a druggable target. Small molecule inhibitors of BCL-2 are in active clinical studies. ABT-199 (venetoclax, RG7601, GDC-0199) has been granted breakthrough designation by FDA for relapsed or refractory CLL with 17p deletion. In this review, we summarized the latest clinical development of ABT-199/venetoclax and other novel agents targeting the BCL-2 proteins.

## Introduction

Tyrosine kinase inhibitors have ushered cancer treatment to the era of targeted therapy [1–7]. Monoclonal antibodies have revolutionized therapy for lymphoma [8–13]. Cancer immunotherapy highlights the latest development in clinical oncology [14–20]. In addition, novel agents/vehicles, such as Bruton's tyrosine kinase (BTK) inhibitors (e.g., ibrutinib) [21–24], chimeric T-cell receptors (e.g., CART19) [25–29], NK cell-specific antibodies (e.g., AFM13) [11, 30, 31], and CD19 BiTE antibodies [32, 33], are in active clinical trials. It has been well documented that B-cell lymphoma-2 (BCL-2) plays a major role in cellular apoptosis and is a druggable target. Several small molecule inhibitors of BCL-2 are in active clinical studies. ABT-199 (venetoclax, RG7601, GDC-0199) has been granted breakthrough designation by FDA for relapsed or refractory chronic lymphoid leukemia (CLL) with 17p deletion. This review focused on the current clinical development of a highly effective class of small molecule BCL-2 inhibitors, including ABT-199/venetoclax.

## BCL-2 gene and BCL-2 proteins

The BCL-2 gene was identified by cloning the breakpoint of a t(14;18) translocation which was found frequently in human B-cell lymphomas [34]. The BCL-2 gene resides on chromosome 18q21.33. The BCL-2 protein has 239 amino acids and a molecular weight of 26 KDa. It was the first identified major apoptotic regulator. The ability to abrogate the death signal is a key hallmark of cancer. BCL-2 plays a major role in tumorigenesis and chemoresistance.

**Fig. 1:**
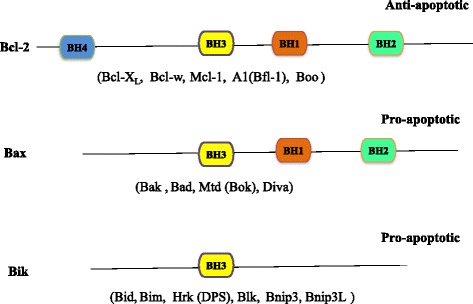
Structures of BCL-2 family proteins. According to the BH domains, the BCL-2 family proteins can be categorized into three subsets. BH4-containing BCL-2 and related BCL-X_L_, BCL-w, MCL-1, A1(BFL-1), and Boo are anti-apoptotic proteins. The remaining two subsets (BAX and Bik subgroups) do not have a BH4 domain and are pro-apoptotic proteins

There are multiple proteins in the BCL-2 family [35] (Fig. 1). The pro-death proteins include BCL-2-associated X protein (BAX), BCL-2 antagonist/killer 1 (BAK), BCL-2-associated agonist of cell death (BAD), BCL-2-like 11 (BIM), NOXA, and BCL-2 binding component 3 (PUMA), whereas the pro-survival proteins include BCL-2, BCL-X_L_, BCL-2-like 2 (BCL-w), myeloid cell leukemia sequence 1 (MCL-1), and BCL-2-related protein A1 (BFL-1).

The roles of BCL-2 family proteins in cellular apoptosis and oncogenesis have been extensively studied [35, 36]. Different members of the BCL-2 family of proteins have pro- and anti-apoptotic functions, with their core function being the regulation of mitochondrial outer membrane permeability [37]. This in turn regulates the release of pro-apoptotic factors such as the second mitochondrial activator of caspases/direct inhibitor of apoptosis protein binding protein with a low isoelectric point (Smac/DIABLO), Omi/HtrA2 [38], apoptosis-inducing factor (AIF), endonuclease G [39], and cytochrome-C [40, 41].

BCL-2 proteins can be classified into three subsets according to the number of BCL-2 homology (BH) domains [42] (Fig. 1). The presence of all four BH domains is the hallmark of all anti-apoptotic BCL-2 proteins, such as BCL-2, BCL-X_L_, and MCL-1, as mentioned above. Pro-apoptotic BCL-2 family proteins typically have three BH domains and are further subdivided into the BAX subset (example: BAX and BAK) and the BH3 subset [example: BH3 interacting domain death agonist (Bid) and BAD] which only share homology at the BH3 domain [43, 44].

BCL-2 directly inhibits the influx of adenine nucleotides through the outer mitochondrial membrane. This reduces ATP hydrolysis and inhibits cytochrome-C release. BAX and BAK act through opposite mechanism and are pro-apoptotic. Other members of the pro-apoptotic pathway also function through the direct release of cytochrome-C or inhibition of BCL-2. Of note, BCL-2 also maintains cells in the G_0_ phase in the absence of survival/growth factors—a potent oncogenic mechanism.

## BCL-2 inhibitors

By taking the advantage of the function of BH3 subset pro-apoptotic proteins in promoting programed cell death, multiple BH3 mimetics have been developed as cancer therapeutics. They interact in an inhibitory manner with the anti-apoptotic proteins BCL-2, BCL-X_L_, and BCL-w.

## ABT-737

ABT-737 is a small molecule inhibitor of BCL-2, BCL-X_L_, and BCL-w [45]. ABT-737 showed in vitro activity against lymphoma and small cell carcinoma cells. Subsequent in vitro studies showed activity against myeloma [46, 47], acute leukemia [48, 49], and lymphoma. Further studies confirmed in vivo activity of ABT-737 in mouse xenograft models [50–53]. However, this compound has low solubility and oral bioavailability.

## ABT-263 (navitoclax)

ABT-263 (navitoclax) is another potent small molecule inhibitor of BCL-2, BCL-X_L_, and BCL-w. It was tested on multiple cell lines in vitro and in xenograft models [54] and shown to have significant activity against acute lymphoblastic leukemia (ALL) cell lines. Subsequent studies showed in vitro activity against leukemia and lymphoma cells [55] with efficacy replicated in mouse models for pediatric ALL [56], non-Hodgkin’s lymphoma [57], and myeloma [58].

Subsequently, phase I trials of ABT-263 were carried out in lymphoid malignancies. The first trial to evaluate ABT-263/navitoclax studied two different dosing schedules in 55 patients with relapsed/refractory (R/R) lymphoid malignancies [59]. The first dosing evaluation consisted of navitoclax given orally once a day at dose ranges from 10 to 440 mg from days 1 to 14 in a 21-day cycle (intermittent schedule). The second dosing schedule consisted of navitoclax once daily at dose ranges from 200 to 425 mg for days 1–21 in a 21-day cycle (continuous schedule). Due to significant thrombocytopenia reported from earlier trials, a loading dose of 150 mg daily for 7–14 days was used for the continuous dosing. The maximal tolerated dose was noted to be 315 mg for the intermittent schedule and 325 mg for the continuous schedule. In the intermittent schedule regimen, at 440 mg, grade III transaminitis and grade IV thrombocytopenia were observed. In the continuous-schedule regimen, grade III transaminitis and gastrointestinal bleed were observed [59]. A phase IIa study is currently ongoing.

Another phase I trial evaluated intermittent and continuous dosing in 29 patients with relapsed/refractory CLL [60]. The patients received oral navitoclax daily either for 14 days (10, 110, 200, or 250 mg/day; *n* = 15) or 21 days (125, 200, 250, or 300 mg/day; *n* = 14) of each 21-day cycle. Dose escalation was done according to a continual reassessment methodology. The phase 2 dosing was determined to be 250 mg daily in a continuous dosing schedule. The dose-limiting toxicity (DLT) was mainly due to dose-related thrombocytopenia. Efficacy data showed partial response in 9 (31 %) of the patients and stable disease in 18 patients (8 with durable SD > 6 months, 7 > 12 months). Reduction in lymphocytosis was seen in 50 % of the evaluable patients. Median progression-free survival was 25 months. Navitoclax was found to have activity in high-risk patients with fludarabine-refractory disease, bulky adenopathy, and del(17p) CLL.

## ABT-199 (venetoclax, RG7601, GDC-0199)

### Preclinical studies of ABT-199

The dose-limiting severe thrombocytopenia from ABT-263/navitoclax quenched the enthusiasm for further clinical development of this compound. Preclinical studies of ABT-737 also revealed decreased platelet survival [61]. A seminal study by Mason et al. [62] had previously highlighted the importance of BCL-X_L_ in platelet survival, its role in the pro-apoptotic activity of BAK, and a gradual reduction in BCL-X_L_ levels culminating in the apoptosis of senescent platelets. The term *molecular clock* was used to describe this temporal switch from anti-apoptosis to pro-apoptosis [62]. Therefore, BCL-X_L_ inhibition could speed up this molecular clock and lead to decreased platelet survival—the mechanism implicated in ABT-737/ABT-263-induced thrombocytopenia.

The need to develop a BCL-2 inhibitor sparing BCL-X_L_ and platelets sparked the discovery and development of ABT-199 (venetoclax) [63]. X-ray complex-based structures determined the presence of high-affinity interactions in two hydrophobic pockets in the three-dimensional structure of anti-apoptotic BCL-2 proteins. These hot spots, named P2 and P4, bind to the 1-chloro-4-(4,4-dimethylcyclohex-1-enyl)benzene and the thiophenyl moiety of navitoclax, respectively. Along with the hydrophobic interactions, electrostatic interactions between arginine residues (on anti-apoptotic proteins) and aspartate residues (on pro-apoptotic molecules) were also underscored. Through reverse engineering of navitoclax, structural modifications were made so that a new molecule was created to have similar hydrophobic interaction but different electrostatic interaction with Arg103 (specific to BCL-2 since BCL-X_L_ has Glu96). This led to the discovery of BCL-2 binding molecule, ABT-199.

ABT-199 (venetoclax) represents the first-in-class, selective, oral BCL-2 inhibitor sparing platelets [63]. It showed sub-nanomolar affinity to BCL-2 (*K*_i_ < 0.010 nM) with antitumor activity against non-Hodgkin’s lymphoma (NHL) [63], CLL [64], and acute leukemias [65, 66] in vitro. In vivo mouse xenograft studies showed activity against aggressive (Myc+) lymphomas [67] as well as acute leukemia [68].

BCL-2 over-expression plays a central role in follicular lymphoma (FL). In vitro studies were done to characterize the effects of ABT-199 in the t(14;18)+ FL cell lines WSU-FSCCL and FC-TxFL2 and in primary FL cells [69]. JNK kinase phosphorylation and inactivation as well as substantial decrease of mitochondrial potential in FC-TxFL2 cells were induced by ABT-199. Similar expression levels were seen in the anti-apoptotic (such as BCL-2, BCL-X_L_, MCL-1) and pro-apoptotic proteins (such as BAX, PUMA, BAK, BAD, NOXA, Bid, Bok) in both cell lines. No significant change was seen in these proteins upon ABT-199 treatment. In this study, the ABT-199 resistant cell line was established. Increased levels of MCL-1 and elevated phosphorylation of BCL-2 on T56 and of AKT on S473 were demonstrated. In addition, increased autophagy was shown in comparison to parental cells. It was also demonstrated in this study that the combination of two epigenetic agents, decitabine and vorinostat, was able to overcome ABT-199 resistance. Even though ABT-199 should be theoretically active in FL as a BH3 mimetic, activity in patients with FL has not been as dramatic as has been observed for CLL. Acquired resistance developed easily in the in vivo study in the xenograft model, which may explain the relatively higher failure possibility in FL than in CLL.

ABT-199 was studied in combinations with tyrosine kinase inhibitors (TKIs), including imatinib, nilotinib, dasatinib, or ponatinib, in cells from six patients with blast-crisis chronic myeloid leukemia (CML) [70]. All six patients were resistant to TKIs, three of them with T315I mutation. This study further revealed in a CML mouse model that ABT-199 alone or in combination was better than nilotinib in eradicating CML stem cells in vivo.

A combination of ibrutinib and ABT-199 was also studied in mantle cell lymphoma (MCL) and CLL [71]. The ibrutinib and ABT-199 combination substantially induced apoptosis of primary cells from MCL and CLL patients compared to each single agent alone (combo: 23 %, ibrutinib: 3.8 %, ABT-199: 3.0 %). When BCL-2 and BTK target genes as well as emergent genes were characterized using a protein-protein network interaction model, synergistic activation of apoptosis genes of p53 and BIM was revealed. Little off-target effect was seen with the combination nor with individual drugs on normal peripheral T cells [71].

Selinexor (KPT-330), a first-in-class selective inhibitor of nuclear export (SINE) agent, has been shown to have antiproliferative and pro-apoptotic activities against MCL and other cancer cells [72–76]. A combination of ABT-199 and selinexor was studied in MCL cell lines [77]. The combination demonstrated synergistic antiproliferative effects through inhibition of mTOR signaling, downregulation of ribosomal biosynthesis, and induction of mitochondria-mediated apoptosis.

MCL-1, an anti-apoptotic protein, is one of the main targets of homoharringtonine (HHT) and omacetaxine, both of which have been in clinical applications [78, 79]. To test the dual inhibition of BCL-2 and MCL-1, HHT and ABT-199 were combined and tested in seven diffuse large B-cell lymphoma cell lines [80]. The study confirmed decreased expression of MCL-1 protein in all the cell lines. It was also demonstrated that high expression of BCL-2 positively correlated with sensitivity to ABT-199, irrespective of expression levels of BCL-X_L_ and MCL-1. BCL-2 and BCL-X_L_ expression levels negatively correlated with sensitivity to HHT. However, the expression level of MCL-1 did not correlate with sensitivity to HHT.

## Clinical studies of ABT-199

Preliminary data from a phase I trial of ABT-199 showed an overall response rate (ORR) of 84 % in 56 patients with relapsed/refractory CLL [81] (Table 1). At a median follow-up of 10.9 months, a complete response (CR) of 23 % and partial response (PR) of 61 % were achieved. ABT-199 dosages of 150–1200 mg given once on days 3 or 7 followed by once daily were used. Diarrhea (46 %), neutropenia (43 %), fatigue (34 %), upper respiratory tract infection (29 %), and cough (25 %) were the most common side effects. Dose-limiting toxicities (DLTs) were reported to include six tumor lysis syndromes (TLSs), one sudden death, and one grade IV neutropenia. TLS complication is now better prevented with a lead-in dose-escalation step [82].

**Table 1:** Single agent clinical trials of venetoclax/ABT-199

Clinical trials	Diseases	Responses	References
Phase I	R/R CLL	ORR—79 %	81, 83
Phase Ia	R/R NHL	ORR—48 %	84
Phase II	R/R AML	ORR—15 %	85
	New AML		

*R/R* relapsed/refractory, *ORR* overall response rate, *CLL* chronic lymphoid leukemia, *NHL* non-Hodgkin’s lymphoma, *AML* acute myeloid leukemia

The latest update showed an ORR of 79 % in 84 patients with relapsed/refractory (R/R) CLL with median duration of response of 20.5 months. The most common adverse events (AEs) remained similar—neutropenia (39 %), diarrhea (36 %), nausea (35 %), upper respiratory tract infection (31 %), and fatigue (27 %). Grade 3/4 AEs included neutropenia, anemia, TLS (8 % including one death), and thrombocytopenia (7 %) [83].

A phase Ia trial of ABT-199 in R/R NHL used continuous daily dosing of 200–900 mg. A single dose was administered on day 7 followed by a lead-in period with stepwise upward titration over 2–3 weeks. An ORR of 48 % was achieved in 40 patients with responses across various NHL subtypes; the most common AEs included nausea (34 %), upper respiratory tract infection (27 %), diarrhea (25 %), and fatigue (21 %). DLTs included subclinical TLS (based on laboratory criteria) [84].

The single-agent ABT-199 was studied in a phase 2, open-label, multicenter trial in patients (pts) with high-risk R/R AML and in untreated patients who were unfit for intensive chemotherapy [85]. The study allowed intra-patient dose escalation when a patient received 20 mg ABT-199 on week (Wk) 1 day 1. Daily escalation was implemented to target a final dose of 800 mg on day 6 and daily thereafter. Those patients without a CR or CR with incomplete hematological recovery (CRi) at the first scheduled assessment (end of Wk 4) were able to escalate to 1200 mg. Of the 32 pts enrolled, 30 (93.8 %) had R/R disease, 12 (37.5 %) had a history of myelodysplastic syndrome, and 2 (6 %) had a history of myeloproliferative neoplasms. The patients were heavily pretreated. Fourteen (44 %) had progressed through at least three prior treatments and 22 (69 %) had failed hypomethylating agents. The most common treatment emergent AEs (TEAEs) (in ≥25 % of patients) were nausea, diarrhea, fatigue, neutropenia, and vomiting. Severe AEs were febrile neutropenia, anemia, and pneumonia. One CR and four CRi were seen at first assessment. Of the five patients with CR/CRi, three had IDH mutation. The ORR was 15.5 % (5/32). Therefore, ABT-199 appears to have considerable single-agent clinical activity in patients with poor prognosis R/R AML. It is worthy of note that patients with IDH mutation may be particularly sensitive to ABT-199.

## ABT-199 in combination regimens

A phase Ib trial of ABT-199 in combination with rituximab (R) in patients with relapsed/refractory CLL enrolled 37 patients. Rituximab dosage was 375 mg/m^2^ titrated to 500 mg/m^2^ monthly for 6 months. ABT-199 dosages began at 20 mg titrated weekly to 200–600 mg. Eighteen patients had completed therapy with 39 % showing CR and 39 % achieving PR (ORR 78 %). Most common AEs included neutropenia (43 %), nausea (38 %), and diarrhea (30 %). DLTs were reported to be thrombocytopenia and hemophagocytic syndrome [86]. An update of the study was reported at 2014 ASH which included 49 pts in five dose-escalation cohorts (*n* = 41) and one safety expansion cohort (*n* = 8) [87]. The patients were heavily pretreated, with a median number of prior therapies at 3 (range 1–10). Thirteen patients (27 %) were refractory to fludarabine and nine (18 %) were R-refractory. Among them also included nine (18 %) with del(17p). The ORR was 88 %, with 11 (32 %) achieving a CR/CRi and 20 (56 %) achieving PR in the 34 pts who were evaluable for response in dose-escalation cohorts. The recommended phase 2 dose (RPTD) of ABT-199 is 400 mg daily. The combination of ABT-199 and R was well tolerated and induced an ORR of 86 % with 31 % CRs in pts with CLL/SLL [87]. A phase 3 trial comparing ABT-199 and R versus bendamustine and R in pts with previously treated CLL is underway (NCT02005471) [88] (Table 2).

**Table 2:** Clinical trials of venetoclax/ABT-199 in combination regimens

Clinical trials	Regimens	Diseases	Responses	References
Phase Ib	V + R	R/R CLL	ORR—88 %	86, 87
			CR/CRi—32 %	
			PR—56 %	
Phase I	V + BR	R/R NHL	ORR—61.5 %	89
Phase Ib	V + BR	R/R CLL	Not reported	90
		Untreated CLL		
Phase Ib	V + O	R/R CLL	Not reported	91
		Untreated CLL		

*R/R* relapsed/refractory, *ORR* overall response rate, *CLL* chronic lymphoid leukemia, *NHL* non-Hodgkin’s lymphoma, *AML* acute myeloid leukemia. For regimens: *V* venetoclax, *R* rituximab, *B* bendamustine, *O* obinutuzumab

A phase I, open-label, multicenter study of ABT-199 in combination with bendamustine (B) and rituximab (R) was reported in patients with R/R NHL [89]. In the dose-escalation portion of the study, ABT-199 doses ranged from 50 to 400 mg. Dosing schedules were as follows: 3, 7, and 28 days/cycle in each 28-day cycle. Standard BR treatment for six cycles were given as the following: B (2 days/cycle, 90 mg/m^2^) and R (1 day/cycle, 375 mg/m^2^). ABT-199 followed by BR was administered on day 1 of cycles 4–6. Upon completion of BR treatment, ABT-199 was continued as monotherapy. At the last update in July 2014, 26 patients have received ABT-199 in combination with BR. Fifteen (57.7 %) patients had follicular lymphoma (FL), eight (30.8 %) had diffuse large B-cell lymphoma (DLBCL), and three (11.5 %) had marginal zone lymphoma (MZL). Out of 26 patients, 11 (42 %) had completed six cycles of BR treatment. The most common TEAEs included nausea (65.4 %), anemia and neutropenia (each in 42.3 %), diarrhea (38.5 %), and thrombocytopenia (34.6 %). Severe AEs included neutropenia (26.9 %) and anemia and thrombocytopenia (each in 19.2 %). DLTs included febrile neutropenia and thrombocytopenia. Dosing schedule was modified to 7 days/cycle for patients in cohort 6. G-CSF prophylaxis was added. No DLTs were observed in the subsequent cohorts. The overall response rate (CR + PR) for ABT-199 + BR was 61.5 % (16/26) for all patients, 73.3 % (11/15) for patients with FL, and 37.5 % (3/8) for patients with DLBCL. Further enrollment employed 400 mg ABT-199 with re-examination of a 28-day cycle schedule with G-CSF prophylaxis in heavily pretreated patients. A phase 2 study (NCT02187861; BO29337) of ABT-199 + BR in R/R FL was planned.

Another phase 1b study evaluated the MTD of ABT-199 in combination with BR in R/R or previously untreated patients (pts) with CLL [90]. Dosages ranged from 100 to 600 mg/day of ABT-199 using a 3 + 3 dose-escalation design. The study also incorporated a gradual dose ramp-up of ABT-199 to reduce the risk of TLS. A preliminary report summarized the data on six pts with R/R CLL, including three pts in the 100-mg cohort and three pts in the 200-mg cohort. TLS risks were considered for risk stratification. The most common AE was anemia with most grade 3 AEs being hematological toxicities. There was no TLS as a result of the ramp-up dosing of ABT-199 and prophylactic measures. Dose escalation in R/R pts was ongoing. Enrollment of previously untreated pts was also planned.

In another phase Ib study, ABT-199 was combined with obinutuzumab in R/R or previously untreated patients with CLL [91]. ABT-199 dosage ranged from 100 to 600 mg/day. A gradual dose ramp-up of ABT-199 and staggering of the two agents were applied to reduce the risk of tumor lysis syndrome (TLS). After completing combination therapy, ABT-199 was continued until disease progression. Nine patients were enrolled in the preliminary report, including three in the 100-mg ABT-199 dosing cohort and six in the 200-mg dosing cohort. Laboratory TLS (characterized by asymptomatic laboratory abnormalities in potassium and phosphate) was observed in 1 of the first 3 patients. One patient had a dose reduction from 100 to 50 mg per day due to prolonged neutropenia but subsequently completed six cycles of combination treatment. For obinutuzumab, infusion-related reactions were seen but limited to the first infusion. The combination was safe and well tolerated. No clinical TLS was observed in these nine patients. Dose escalation of ABT-199 was done in those R/R patients at 400 mg/day.

The phase II trial of ABT-199 in 17p (-) relapsed/refractory CLL patients (NCT01889186 [92]) is underway. Apart from this, multiple studies evaluating ABT-199 in multiple myeloma (NCT01794507, NCT01794520), CLL (NCT02242942, NCT01685892, NCT01671904, NCT02427451, NCT02141282, NCT02401503), and NHL (NCT02187861, NCT02558816, NCT02055820, NCT02471391, NCT02419560, NCT01594229) are currently active.

## Conclusion and future directions

ABT-199/venetoclax showed high response rate as a single agent in refractory/relapsed CLL. In particular, high CR rate was reported in refractory/relapsed CLL (29 % as single agent, 39 % in combination with rituximab). This is quite different from ibrutinib, which acts slowly and rarely induces CR as a single agent in this population of patients. Resistance in lymphoid cells to ABT-199 has been reported [93]. Combination regimens to counteract these mechanisms are being evaluated [94, 95]. The lack of BCL-X_L_ inhibition with ABT-199 significantly reduced the dose-limiting thrombocytopenia. Since BCL-2 plays a major role as an anti-apoptotic protein in multiple hematological malignancies, ABT-199 has the potential to be effective as a single agent or in combination with other agents toward a broad spectrum of hematological malignancies. ABT-199 therefore may lead to the shift of current treatment paradigms in hematological cancers.
